# Relationships between Uncoupling Protein Genes *UCP1*, *UCP2* and *UCP3* and Irisin Levels in Residents of the Coldest Region of Siberia

**DOI:** 10.3390/genes13091612

**Published:** 2022-09-08

**Authors:** Alena A. Nikanorova, Nikolay A. Barashkov, Vera G. Pshennikova, Nyurgun N. Gotovtsev, Georgii P. Romanov, Aisen V. Solovyev, Sargylana S. Kuzmina, Nikolay N. Sazonov, Sardana A. Fedorova

**Affiliations:** 1 Laboratory of Molecular Genetics, Yakut Science Centre of Complex Medical Problems, 677000 Yakutsk, Sakha Republic (Yakutia), Russia; 2 Laboratory of Molecular Biology, M.K. Ammosov North-Eastern Federal University, 677058 Yakutsk, Sakha Republic (Yakutia), Russia

**Keywords:** irisin, uncoupling protein genes, *UCP1*, *UCP2*, *UCP3*, thermoregulation, browning, cold climate, adaptation

## Abstract

Currently, it is known that irisin can participate in the processes of thermoregulation and browning of adipose tissue, and, therefore, it is possible that it is involved in the microevolutionary mechanisms of adaptation to a cold. The aim of this study is to investigate the relationship between the uncoupling protein genes (*UCP1, UCP2*, *UCP3)* and the irisin levels in the residents of the coldest region of Siberia. The sample consisted of 279 Yakut people (185 females, 94 males, average age 19.8 ± 2.03 years). The females plasma irisin concentration was 8.33 ± 2.74 mcg/mL and the males was 7.76 ± 1.86 mcg/mL. Comparative analysis of irisin levels with the genotypes of six studied SNP-markers in females revealed a significant association of irisin with rs1800849-*UCP3.* The TT genotype of rs1800849 was associated with elevated levels of irisin (*p* = 0.01). It was also found that this TT genotype in females was associated with reduced weight and height (*p* = 0.03). We searched for natural selection signals for the T-allele rs1800849-*UCP3*; as a result of which, it was found that this allele has a significantly high frequency of distribution in northern (45%, CI: 0.42–0.484) compared with southern Asian populations (28%, CI: 0.244–0.316) (*p* = 0.01). The results obtained indicate the probable involvement of irisin and the *UCP3* gene in thermoregulation, and the spread of its allelic variants is probably related to adaptation to a cold climate.

## 1. Introduction

Thermoregulation is one of the main physiology mechanisms in warm-blooded organisms. The ability to deal with cold stress is crucial for survival. When ambient temperature decreases, adaptive (also known as facultative) thermogenic mechanisms are activated to maintain optimal body temperature and normal functioning of the organism. In response to cold, the shivering thermogenesis activates first [1]. Shivering is an iterative process of the reduction and relaxation of skeletal muscle, activated by stimulation of the neuromuscular junction, which activates the hydrolysis of ATP with the release of heat [2]. However, this type of thermogenesis is considered as short-term, since it can lead to damage of the skeletal muscles [3]. Therefore, with prolonged cold stress, nonshivering thermogenesis in brown adipose tissue (BAT) and, to a lesser degree, in white adipose tissue (WAT) is activated [4,5]. Recent studies suggest that BAT-mediated thermogenesis may play one of the main roles in energy balance [6].

Nonshivering thermogenesis is related by the uncoupling protein 1 (UCP1), which, in turn, reduces the proton gradient in oxidative phosphorylation and weakens the work of mitochondrial ATP synthase, resulting in increased heat production [5]. In addition, it is currently known that nonshivering thermogenesis can occur in beige adipocytes, originating from a subpopulation of white adipocytes during the browning process, which are found as inclusions in white adipose tissue [7,8,9,10]. Prolonged exposure to cold temperatures results in browning, and the process is currently being investigated by many studies around the world [7,11,12,13,14,15,16,17,18,19]. In addition to UCP1, several other uncoupling proteins are known. The uncoupling proteins (UCP1–UCP3) of the mitochondria in brown adipose tissue are specific components unique to mammalian cells. UCP1 diverts energy from ATP synthesis to thermogenesis in the mitochondria of brown adipose, by catalyzing a regulated leak of protons across the inner membrane. UCP2 and UCP3 are present, though with much lower abundance than UCP1. The main function of UCP2 is the control of mitochondria-derived reactive oxygen species. UCP3 was handled for a long time as a twin of UCP2, due to its very high homology and the history of its discovery. However, the exact roles of *UCP2* and *UCP3* in thermoregulation are not fully known [20,21,22].

Irisin is a myokine and is produced by the FNDC5 protein (Fibronectin Type III Domain Containing 5), in response to the activation of the γ coactivator 1 of the α receptor activated by the proliferator peroxisome (PGC-1 α) [23]. While irisin is primarily known as a myokine [23], it is also released from adipose tissue (adipo-myokine) [19,24]. Studies have also shown that irisin is foremost released in response to physical activity [23,25] and from muscle-shivering-related cold exposure, serving to augment brown fat thermogenesis [23,26]. Irisin can stimulate the browning of white adipocytes, which increases the expression of UCP1, increasing energy expenditure and improving glucose tolerance in vivo and in vitro [23,27]. Thereby, it may be assumed that irisin plays a role in the energy metabolism and thermogenesis of indigenous people living in cold environmental conditions. The Sakha Republic is the largest region of Siberia as well as the subarctic and Arctic regions. The climate of the republic is characterized as sharply continental, with a long winter period and a short summer. The recorded minimal air temperature in this Siberian region is −71.2 °C [28]. The Yakuts (Sakha) are one of the numerous indigenous peoples of the Sakha Republic (466,492 people, according to the Russian Census, 2010). The indigenous peoples of Siberia (including the Yakuts) have developed certain physiological and metabolic features to adapt to the climatic conditions: higher levels of energy metabolism, lower lipid levels in blood serum [29], higher blood pressure levels [4,29,30,31,32], and seasonal variation in free thyroid hormones in the blood [33]. Thus, we assume that the Siberian peoples may have developed genetic features regulating blood irisin levels as an adaptation to the cold climate.

Consequently, the aim of this study is to identify the relationship between uncoupling protein genes (*UCP1, UCP2*, *UCP3*) and the irisin blood levels in the Yakut population living in the coldest region of Siberia.

## 2. Materials and Methods

### 2.1. Subjects

The study sample was comprised of 279 Yakut individuals: 185 females and 94 males (mean age 19.8 ± 2.03 years). They presented no health issues at the time of the study and had completed a questionnaire in which they specified their ethnicity, age, and sex. All participants gave written informed consent for participation in the study. This study was approved by the local Biomedical Ethics Committee at the Yakut Scientific Center of Complex Medical Problems, Siberian Branch of the Russian Academy Scientific of Medical Sciences, Yakutsk, Russia (Yakutsk, Protocol No. 16, 13 December 2014).

### 2.2. Anthropometric Measurements

Anthropometric data (height in centimeters, body weight in kilograms) were measured for all participants by standardized methods. Body mass index (BMI) was calculated by dividing body mass by the square of the body height. The sample was divided into three groups by BMI [34]: normal weight (18.5–24.99 kg/m^2^), underweight (≤18.49 kg/m^2^), and overweight/obese (≥25 kg/m^2^).

### 2.3. Plasma Irisin Analyses

Fasting plasma irisin levels (mcg/mL) were determined with the human irisin sandwich enzyme-linked immunoassay (ELISA) “Irisin ELISA BioVendor” (BioVendor–Laboratorni medicina A.S., Czech Republic, Brno). The concentration of irisin in the samples was measured at the wavelength of 450 nm on a VICTOR X5 Multilabel Plate Reader (Perkin Elmer Inc., Waltham, MA, USA).

### 2.4. DNA Analysis

DNA was isolated from the blood using phenol–chloroform method. A total of 6 polymorphisms of uncoupling protein genes were genotyped using the PCR-RFLP method (polymerase chain reaction–restriction fragment length polymorphism). Polymerase chain reaction was performed on a BioRad T100 Thermal Cycler (Bio-Rad Laboratories, Inc., Hercules, CA, USA). The data, including the PCR product size, primer sequence, annealing temperature, and restriction enzymes, are given in Supplementary Table S1 [35].

### 2.5. Search for Signals of Natural Selection

Data from the database “1000 Genomes Project” [36] and Stepanov et al. [37] were used in the search for possible indicators of natural selection for cold climate adaptation. Data were extracted for the following 28 populations: Nivkhs (Russia), Koryaks (Russia), Chukchi (Russia), Buryats (Russia), Khanty (Russia), Kets (Russia), Esan (Nigeria), Luhya (Webuye, Kenya), Gambians (Gambia), Mende (Sierra Leone), Yoruba (Ibadan, Nigeria), Britons (England and Scotland), Finns (Finland), Iberians (Spain), Tuscans (Italy), Punjabis (Lahore, Pakistan), Bengalis (Bangladesh), Gujarati Indian (USA), Indian Telugu (United Kingdom), Sri Lankan Tamils (United Kingdom), Han Chinese (Beijing, China), Chinese Dai (Xishuangbanna, China), southern Han Chinese (China), Japanese (Tokyo, Japan), Vietnamese (Ho Chi Minh City, Vietnam), Colombians (Medellín, Colombia), Puerto Ricans (Puerto Rico), and Peruvians (Lima, Peru). Thus, the total sample size comprised 1300 individuals. Using Surfer 12.0 software (Golden Software, Golden, CO, USA), a map of the allele frequency distribution in populations of North and South America, Africa, and Eurasia was composed, which included data on the allele frequencies of these 1300 individuals.

### 2.6. Statistical Analysis

The received data were analyzed using Statistica 13.5 (TIBCO Software Inc., Palo Alto, CA, USA). Values of *p* ≤ 0.05 were considered statistically significant. Quantitative results are reported as the mean ± standard deviation. The Kolmogorov–Smirnov test was performed to test the normal distribution and homogeneity of the examined data. The association of BMI with irisin levels was assessed with the correlation analysis. Comparative analysis of the three BMI groups between females and males was performed with a Mann–Whitney U test for the underweight and overweight/obese groups and with a Student’s t-test for the individuals with normal weight. To identify statistically significant associations between the genotypes of the 6 SNPs of the uncoupling protein *UCP1*, *UCP2*, and *UCP3* genes and plasma irisin concentrations, a one-factor analysis of variance (ANOVA) was performed. A comparative analysis of the average levels of irisin, BMI, weight, and height in females for rs1800849 of the *UCP3* gene was performed using a Mann–Whitney U test. Statistical analysis of the frequencies of T-allele of the rs1800849 of the *UCP3* gene was performed using the Sampling program, kindly provided by M. Macaulay and M. Metspalu. Differences at the 95% significance level were considered statistically significant.

## 3. Results

### 3.1. Plasma Irisin Levels

**Figure 1:**
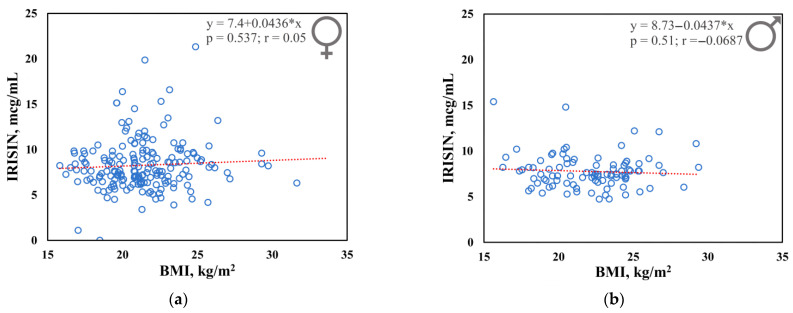
Correlation analysis of BMI and plasma irisin levels in females (**a**) and males (**b**).

The plasma irisin concentration was 8.33 ± 2.74 mcg/mL in females and 7.76 ± 1.86 mcg/mL in males. There was no statistically significant association of plasma irisin levels with BMI in either females (*p* = 0.537; *r* = 0.05) or males (*p* = 0.51; *r* = −0.0687) (Figure 1). Data presented in the study are provided in Supplementary Table S2.

Table 1 presents irisin levels and anthropometric characteristics of the sample (*n* = 279), stratified by BMI into three groups—underweight, normal weight, and overweight/obese. Males displayed significantly higher height and weight than females (*p* = 0.01) in all groups. Males with a normal weight displayed a significantly higher BMI than females (*p* = 0.03). In the normal weight group, statistically significant differences in irisin levels were detected (*p* = 0.02).

### 3.2. Association Analysis between Irisin Levels and Six Single Nucleotid Polymorphisms of the Uncoupling Protein Genes

**Figure 2:**
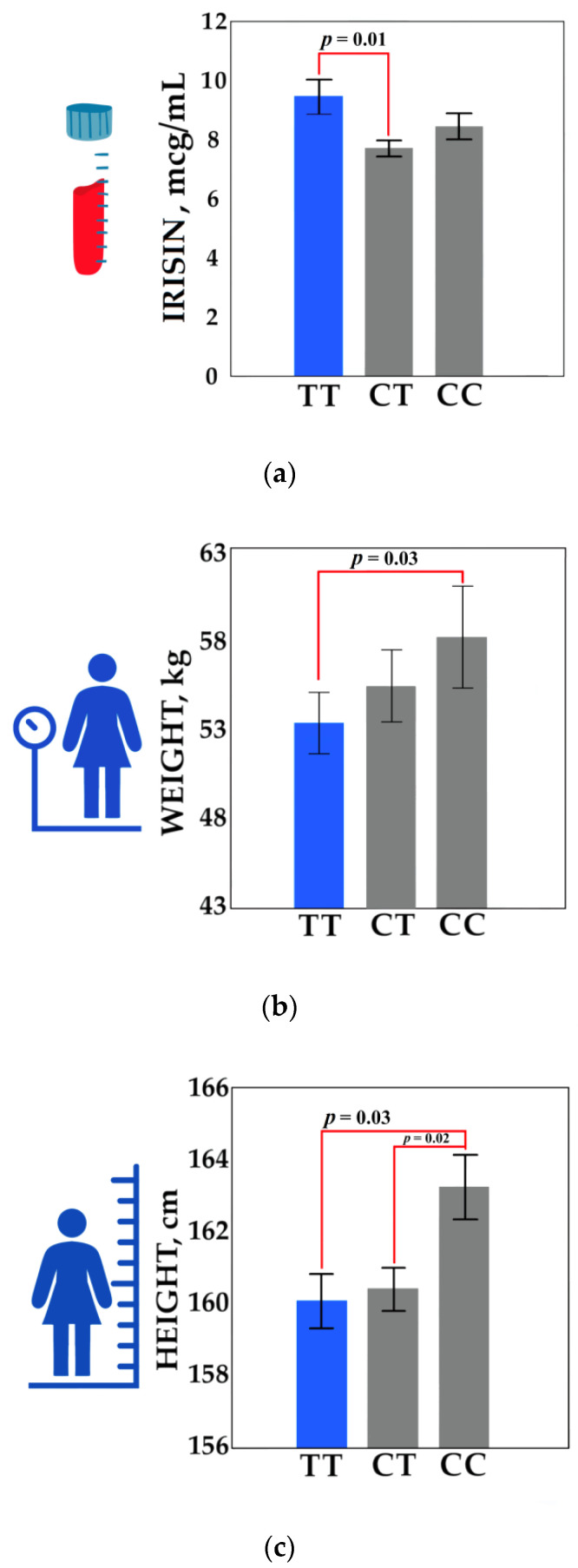
Irisin levels, weight, and height divided by the rs1800849 (*UCP3*) genotypes: (**a**) comparison of irisin levels for the rs1800849 genotypes for the group of females with normal BMI (*n* = 144); (**b**) comparison of weight for the rs1800849 genotypes for all females (*n* = 185); (**c**) comparison of height for the rs1800849 genotypes for all females (*n* = 185). A comparative analysis was performed using a Mann–Whitney U test. Values of *p* ≤ 0.05 were considered statistically significant. Values are shown as mean ± SEM.

The allele frequencies and the genotypes of the six single nucleotide polymorphisms of the uncoupling proteins genes (*UCP1, UCP2, UCP3*), which are potentially related to processes of thermogenesis, are given in Supplementary Table S3. Comparative analysis of irisin levels with the genotypes of the six SNP variants of the uncoupling protein genes were performed separately for females and males with normal weight (Supplementary Table S4). As the result, in males there was no association between irisin levels and the studied genotypes. Irisin levels, weight, and height divided by the rs1800849 (*UCP3*) genotypes are presented in Figure 2.

In females, association between irisin levels and studied genotypes were found for rs1800849 (*UCP3*). For rs1800849 (*UCP3*), irisin levels were higher in the TT homozygotes (9.47 ± 3.77 mcg/mL) compared to the CT heterozygotes (7.73 ± 2.24 mcg/mL) (*p* = 0.01) (Supplementary Table S4). An additional analysis was performed in order to identify the relationship of BMI, height, and weight, with the genotypes of the studied SNP (rs1800849–*UCP3*) in all females. Associations of the TT genotype with reduced weight and height were revealed. In females with the TT genotype, weight (53.61 ± 6.97 kg) and height (160.04 ± 5.85 cm) were significantly lower than in the homozygotes for the CC genotype (weight 58.27 ± 11.56 kg, *p* = 0.03; height 163.18 ± 5.9 cm, *p* = 0.03) (Supplementary Table S5). However, we can say that there is a certain tendency towards a reduction in BMI in females with the TT genotype (Supplementary Table S5). Thus, it can be said that the polymorphism of rs1800849 (*UCP3*) can affect anthropometric parameters (BMI, weight, height) and irisin levels in the blood.

### 3.3. Search for Signals of Natural Selection for Cold Climate Adaptation for rs1800849 of the UCP3 Gene

**Figure 3:**
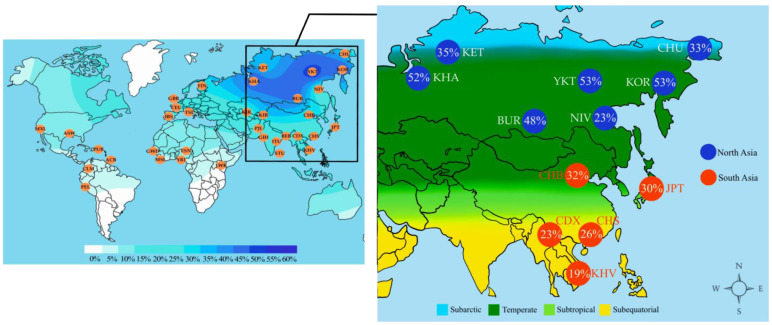
Geographical distribution of T-allele frequency of rs1800849 (*UCP3*). Allele frequency gradations are indicated on the color scale. CZ—climatic zone; “North Asia” [33]: YAK –Yakuts (this study), CHU—Chukchi, KOR—Koryaks, KET—Kets, KHA—Khanty, BUR—Buryats, NIV—Nivkhs; “South Asia” [32]: JPT—Japanese, Tokyo, Japan; CHB—Han Chinese, Beijing, China; CHS—Southern Han Chinese, China; CDX—Chinese Dai, Xishuangbanna, China; KHV—Kinh, Ho Chi Minh City, Vietnam.

The single nucleotide polymorphism of the *UCP3* gene (rs1800849) that was identified to be associated with irisin levels in the Yakut population was analyzed for possible signals of natural selection towards cold-climate adaptation. We used the “1000 Genomes Project” [36] database and published studies [37] for comparative analysis of the prevalence of the polymorphisms in 12 East Asian populations from different climatic zones (Supplementary Table S6). Seven populations of Siberia (Yakuts, Nivkhs, Koryaks, Chukchi, Buryats, Khanty, Kets) living in subarctic and temperate climates were included in the “North Asia” group. Other East Asian populations from temperate (Han Chinese—CHB), subtropical (Japanese—JPT; southern Han Chinese—CHS; Chinese Dai—CDX), and subequatorial (Vietnamese–KHV) climate zones were combined into the “South Asia” group. The prevalence of the T-allele of rs1800849 (*UCP3*) was found to be significantly higher in the “North Asia” group (45%, CI: 0.42–0.484) compared to the “South Asia” group (28%, CI: 0.244–0.316) (*p* = 0.01) (Figure 3).

## 4. Discussion

In this study, we analyzed the plasma irisin levels and data for six SNPs of uncoupling protein genes (*UCP1*, *UCP2*, *UCP3*) that are possibly involved in thermogenesis processes [38,39,40,41,42,43,44,45,46] in the residents of the coldest region of Eastern Siberia (Yakut population). In the Yakut population, irisin levels were found to be independent of BMI (females *p* = 0.537; males *p* = 0.51). Moreover, in the normal weight group, we found a significantly higher irisin levels in females (*p* = 0.02) compared to males. Such differences were not revealed in the groups with deficient and excessive weight. However, the results of other studies on the relationship of irisin with sex and with BMI are too contradictory to draw certain conclusions [25,47,48,49,50,51,52,53,54,55,56,57,58,59,60,61]. Therefore, further research is required in order to define the influence of sex and anthropometric indicators on the blood irisin levels.

Comparative analysis of irisin levels and the studied SNP markers demonstrated an association with the rs1800849 polymorphism of the *UCP3* gene. In females with the TT genotype, irisin levels (9.47 ± 3.77 mcg/mL) were significantly higher (*p* = 0.01) compared to those of the CT genotype (7.73 ± 2.24 mcg/mL). In addition, it was found that females with the TT genotype have a smaller height and weight than females with the CC genotype (on average, females with the TT genotype are 3.14 cm shorter and weigh 4.66 kg fewer) (Figure 2). Thus, we can assume that female carriers of the TT genotype are at risk of a delay in growth and being underweight. The effect of the rs1800849 of the *UCP3* gene on BMI and obesity is still being discussed, as there are conflicting research results [62,63]. However, the association of rs1800849 polymorphism with height was found in children from China, where carriers of TT and CT genotypes had a lower height compared to CC carriers [62]. In Pima Indians, T-allele was associated with increased expression of UCP3 mRNA in skeletal muscles [63], and the expression level negatively correlated with BMI [64]. It can be assumed that carriers of the TT genotype (rs1800849) have an increased expression of UCP3 in skeletal muscles, and this overexpression suppresses physical development (lower weight and height). This effect somewhat can be explained because rs1800849 (-55C>T) is located in the promoter region, at 6 bp from the TATA-box, and this location suggests a possible effect on the transcription of the *UCP3* gene [65].

**Figure 4:**
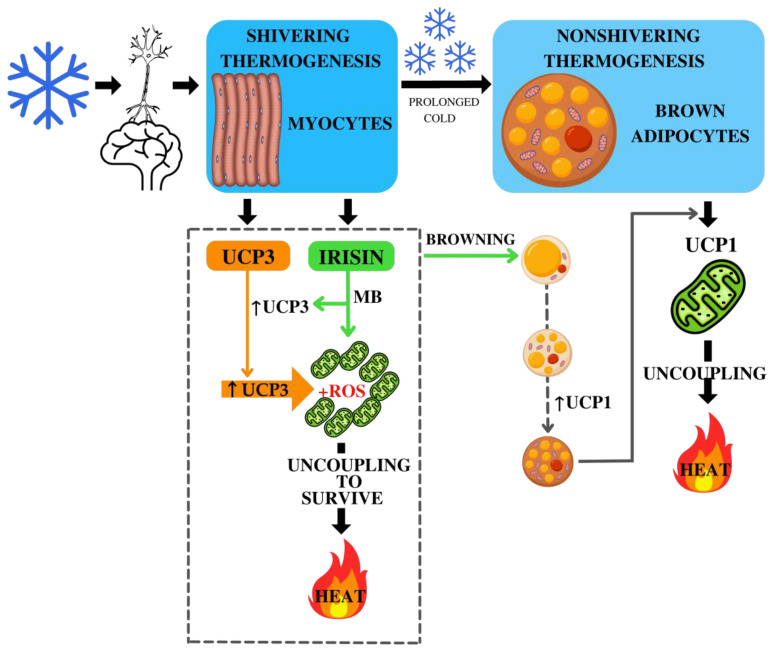
Possible mechanisms of action of irisin in shivering and nonshivering thermogenesis. Note: the dotted rectangle is a possible mechanism based on other studies [67,68,69,70,72,77]. Shivering thermogenesis: when exposed to cold on the body, shivering thermogenesis in skeletal muscles is primarily activated. With this thermogenesis, myocytes begin to release irisin and express UCP3. In turn, irisin acts in three directions: increasing the expression of UCP3, activating mitochondrial biogenesis (MB), and browning. Further, UCP3 participates in the protection of mitochondria from reactive oxygen species (ROS) and produces a soft separation in which the resulting energy is released as heat. Nonshivering thermogenesis: with prolonged exposure to low temperatures, nonshivering thermogenesis is activated in brown adipocytes, with the participation of uncoupling protein-1 (UCP1). Irisin-activated browning of white adipocytes leads to a longer use of nonshivering thermogenesis, to maintain optimal body temperature under cold exposure.

The *UCP3* gene (*SLC25A9*) is located in chromosome 11 (11q13.4), contains 7 exons, and encodes the mitochondrial uncoupling protein 3, which is mainly expressed in skeletal muscles and in BAT [66,67,68,69], and its expression may increase during exposure to cold conditions [70]. The main function of the mitochondrial protein UCP3 is considered to be proton transport, in the presence of fatty acids [71,72]. UCP3 may also play a protective function, by inhibiting the action of reactive oxygen species (ROS) in mitochondria [73,74]. Previously, the association of rs1800849 (-55C>T) with atmospheric air temperature has been demonstrated [37,40] and it is possible that variations in the *UCP3* gene are implicated in resistance to cold [40]. Therefore, our study suggests that the distribution of allelic variants of the *UCP3* gene is probably related to human adaptation to a cold condition. However, the accurate role of *UCP3* in thermoregulation is not fully known, and, perhaps, its thermoregulatory actions are dependent on irisin. Currently, it is already known that irisin can increase the expression of UCP3 in skeletal muscles. In a study on rats, it was shown that the administration of exogenous irisin increases the expression of UCP3 mRNA in muscles and UCP1 in WAT and BAT [75]. In an in vitro study by Vaughan et al. [76], it was found that trypsin-treated myocytes demonstrated enhanced oxidative metabolism and mitochondrial biogenesis and increased expression of UCP3. Therefore, irisin and UCP3 may play an important role in shivering thermogenesis [77]. Thus, when exposed to low temperatures, shivering thermogenesis is activated in the body, which is accompanied by a contraction of skeletal muscles, during which irisin is released and UCP3 expression begins. It is possible that irisin simultaneously induces mitochondrial biogenesis in muscles and browning (in which UCP1 expression increases), and UCP3 performs two functions: proton transport and protection of mitochondria from ROS. ROS are discussed in the regulation of thermogenesis [20,21,22]. Meanwhile, stress and inflammation have been linked to the activation of the tryptophan (Trp)–kynurenine (KYN) metabolic system [78]. According to some researchers, revealing the link between mitochondrial biogenesis and the KYN metabolic system may be a promising option [78]. Experimental data have shown that ROS production is increased in mice with knockout *UCP3* [79,80]. Since irisin can stimulate mitochondrial biogenesis [77], in which the number of mitochondrial copies in myocytes increases to produce a large volume of ATP, the amount of ROS increases and lipid peroxidation processes are activated. The uncoupling protein UCP3 inhibits the production of ROS in mitochondria, by reducing the mitochondrial membrane potential [74]. At the same moment, by reducing the mitochondrial membrane potential, the transfer of electrons in the electron transport chain can be accelerated, and the likelihood of electrons being directly transferred to O_2_ can be minimized [81]. Consequently, mild uncoupling occurs as a feedback mechanism adopted by the body to prevent excessive ROS in the mitochondria, which was termed “uncoupling to survive” [82] (Figure 4). Thus, we can suggest that females with the TT rs1800849 (−55C>T) genotype of the *UCP3* gene might have a greater thermal effect from shivering thermogenesis, to protect the body from cold.

### Limitations of the Study

Although the strength of this study is its investigation of indigenous people of the coldest region of Siberia, who have most likely developed certain metabolic and physiological features to adapt to the climatic conditions, the main limitation is the absence of comparative data of *UCP3* gene variants and irisin levels in peoples of other climatic regions. Since this is a first study about the association between irisin levels with the *UCP3* gene, further extensive studies in different worldwide populations are needed. In addition, our study is limited by the applied research methods based on the associative analyses between SNPs variants of the uncoupling protein genes and irisin levels. For deeper understanding of the relationship between UCPs and irisin in cold tolerance, other experimental studies are needed.

## 5. Conclusions

In this study, we found sex differences in irisin levels in the group of people with normal weight, where females had an increased level of irisin in contrast to males (*p* = 0.02). In the other two groups (underweight and overweight/obesity), no sex-related differences were found (*p* > 0.05). Most likely, this is due to the presence of certain metabolic features in the people from these two groups, which affect the levels of irisin in the blood, or it is due to the small sample of these two groups. Therefore, the sex-related reasons for the difference in irisin levels found in the present work should be investigated in detail in future studies.

We found that in females, the TT genotype of the rs1800849 polymorphism of the *UCP3* gene is associated with increased irisin levels and reduced weight and height. The relationship of the TT genotype of the rs1800849 polymorphism with weight and height may be a consequence of the irisin–*UCP3* association, but this requires further study. At the moment, we can only say that the TT genotype in the Yakuts’ females is associated with the risk of a delay in growth and underweight.

Further analysis of worldwide data showed that the T-allele of rs1800849 (*UCP3*) has a significantly high frequency of distribution in northern Asian populations (45%, CI: 0.42–0.484), compared with southern Asian populations (28%, CI: 0.244–0.316) (*p* = 0.01). These results support the previously stated assumption about the possible association of the rs1800849 polymorphism of the *UCP3* gene with human adaptation to a cold climate.

Finding relationships between *UCP3* and irisin levels can be explained by the possible mechanisms of action of irisin in thermogenesis. We assume that irisin simultaneously induces mitochondrial biogenesis in muscles and browning in which uncoupling proteins expression increases, including UCP3, which is involved in the process of “uncoupling to survive” and the protection of mitochondria from reactive oxygen species, producing a soft separation in which the resulting energy is released as heat. However, this assumption requires experimental confirmation.

## Figures and Tables

**Table 1:** Irisin levels and anthropometric data of the subjects, stratified by BMI and sex.

Characteristics	Underweight (*n* = 36)		*p* ^1^	Normal Weight (*n* = 214)		*p* ^2^	Overweight and Obese (*n* = 29)		*p* ^1^
	F (*n* = 25)	M (*n* = 11)		F (*n* = 144)	M (*n* = 70)		F (*n* = 16)	M (*n* = 13)	
Weight (kg)	44.88 ± 3.71	50.45 ± 3.42	**0.01**	55.53 ± 5.8	66.19 ± 7.44	**0.01**	72.75 ± 11.13	81.46 ± 8.3	**0.01**
Height (cm)	160.24 ± 5.14	170.36 ± 5.89	**0.01**	160.92 ± 6.03	173.33 ± 5.98	**0.01**	162.19 ± 4.96	174.69 ± 6.64	**0.01**
BMI (kg/m^2^)	17.45 ± 0.73	17.39 ± 0.91	0.868	21.42 ± 1.62	22 ± 1.89	**0.03**	27.56 ± 2.88	26.64 ± 1.49	0.539
Irisin (mcg/mL)	7.88 ± 1.96	8.52 ± 2.64	0.904	8.42 ± 2.92	7.51 ± 1.61	**0.02**	8.27 ± 1.96	8.48 ± 2.16	0.965

**Note.** ^1^ Mann–Whitney U test; ^2^ Student’s t-test; F—females; M—males. Data are presented as mean ± std. dev. Statistically significant differences highlighted are bold.

## Data Availability

The data presented in this study are available on request from the corresponding author.

## Supplementary Materials

The following supporting information can be downloaded at: https://www.mdpi.com/article/10.3390/genes13091612/s1. Table S1: List of primers sequences, annealing temperature, and allelic profiles of the studied SNPs. Table S2: Anthropometric characteristics and results of genotyping and ELISA analysis of irisin in 279 individuals in a random sample of Yakuts. Table S3: Allele frequencies of studied SNP-markers of *UCP1, UCP2*, and *UCP3* genes in the Yakut population. Table S4: ANOVA analysis of plasma irisin levels depending on genotypes in Yakuts with normal weight (*n* = 214). Table S5: Analysis of irisin levels, weight, height, and BMI depending on genotypes in the women’s SNP rs1800849 gene, *UCP3*. Table S6: Frequencies of the T allele of the rs1800849 polymorphism (*UCP3*) in 12 Asian populations living in different climatic zones.
