# Supplementary material for: Relationships between Uncoupling Protein Genes UCP1, UCP2 and UCP3 and Irisin Levels in Residents of the Coldest Region of Siberia
Source: Genes (Basel). 2022 Sep 8;13(9):1612. doi: 10.3390/genes13091612 (PMC9498418; doi:10.3390/genes13091612)
Supplement: Supplementary file 1 [file genes-13-01612-s001.zip › genes-1894825-supplementary.pdf]

Supplementary Materials

Table S1. List of primers sequences, annealing temperature and allelic profiles of the studied SNPs

Gene	Chromosomal region	SNP	Primer sequence	Annealing temperature/ time, product size	Restriction enzymes, allele sizes (bp)
UCP1	4q31.1	rs1800592<br>-3826A>G	F: 5'-ACATTTTGTGCAGCGATTTCTG-3'<br>R: 5'-TTCACCACTTCTGACAGGCT-3'	56°C/ 45 sec., 301 bp	*Ksp22I,*<br>A = 265+36<br>G = 301
		rs3811787<br>-412A>C	F: 5'-CCTTCTGTCACCCTTTGGCTGCACACCTTCGCC-3'<br>R: 5'- TGACAAGTTCAGAGTGCTCTT-3'	57°C/ 45 sec.,<br>296 bp	*Bst2UI,*<br>T = 262+34<br>G = 296
UCP2	11q13.4	rs659366<br>-866G>A	F: 5'-AGCGTGACCTCACGCTCCTA-3'<br>R: 5'-GACTGAACGTCTTTGGGACTCCGT-3'	59°C/ 45 sec., 299 bp	*BspFNI,*<br>T = 178+121<br>C = 299
		rs660339<br>p.(Ala55Val)	F: 5'-TTGACAGAATCATACAGGCCGA-3'<br>R: 5'-TTGGAGCATCGAGATGACTG-3'	53,8°C/ 45 sec., 392 bp	*Bst4CI,*<br>G = 392<br>A = 153+110
UCP3	11q13.4	rs1800849<br>-55C>T	F: 5'-CCTTGTCACCAAGGAAGCGTCCACAGCTT-3'<br>R: 5'-CTTCTGGCTTGGCACTGGTCTTATACACCC-3'	59°C/ 45 sec., 215 bp	*SmaI,*<br>C = 185+30<br>T = 215
		rs2075577<br>p.(Tyr210Tyr)	F: 5'-GGGACTGGAACCCAAGTCT-3'<br>R: 5'-ACGACATCCTCAAGGAGAAGCTGCTGGAGTA-3'	58°C/ 45 sec., 249 bp	*RsaNI,*<br>G = 218+32<br>A = 249

Table S2. Anthropometric characteristics and results of genotyping and ELISA analysis of irisin in 279 individuals in a random sample of Yakuts

No	# DNA	Gender	Height, cm	Weight, kg	BMI, kg/m²	Age, years	Irisin, mcg/ml	*UCP1* rs1800592	*UCP1* rs3811787	*UCP2* rs659366	*UCP2* rs660339	*UCP3* rs1800849	*UCP3* rs2075577
1	201	F	168	55	19.49	18	5.64	AA	TT	CT	GA	TT	AA
2	204	F	167	62	22.23	18	4.54	AA	TT	CT	GA	CT	GA
3	205	F	152	48	20.78	19	7.63	AA	GT	CC	GG	CT	GA
4	206	F	155	51	21.23	19	6.17	GA	GT	CT	GA	CT	AA
5	207	F	165	65	23.88	18	9.92	GA	TT	CT	GG	CT	GA
6	208	F	160	56	21.88	18	6.93	GA	TT	CC	GG	TT	AA
7	210	F	168	61	21.61	17	9.92	GA	GT	CT	GA	CT	GA
8	211	F	173	58	19.38	19	6.239	GG	GT	TT	AA	CC	GA
9	213	F	176	110	35.51	18	6.77	GG	GT	TT	AA	CC	GA
10	214	F	169	55	19.26	19	5.854	GA	GG	CT	GA	CT	GA
11	216	F	157	55	22.31	19	7.39	AA	GG	CT	GG	CT	GA
12	217	F	165	65	23.88	18	10.1	GA	GG	TT	AA	TT	AA
13	219	F	158	60	24.03	19	7.273	GG	GG	TT	AA	TT	AA
14	220	F	160	60	23.44	19	3.902	AA	GT	CT	GA	TT	GA
15	223	F	161	57	21.99	18	9.701	AA	TT	CT	GA	TT	AA
16	226	F	159	54	21.36	21	9.61	AA	TT	TT	AA	TT	AA
17	227	F	174	75	24.77	19	9.53	GA	GT	CT	GA	CC	GG
18	228	F	164	51	18.96	20	7.6	AA	GT	CT	GA	TT	AA
19	232	F	158	52	20.83	18	10.7	AA	TT	CT	GA	CC	GG
20	233	F	168	48	17.01	18	6.46	AA	TT	TT	AA	CC	GA
21	234	F	160	57	22.27	19	6.122	AA	GT	TT	AA	CT	AA

22	236	F	150	45	20.00	19	10.37	GA	GT	TT	AA	TT	AA
23	238	F	151	48	21.05	19	6.93	GA	GT	CC	GG	TT	AA
24	239	F	160	53	20.70	19	6.169	AA	TT	TT	AA	CC	AA
25	240	F	165	45	16.53	19	8.01	GA	GT	CT	GA	TT	AA
26	242	F	157	43	17.44	19	7.63	GA	GG	CC	GG	CT	GA
27	243	F	160	54	21.09	19	7.099	GA	GT	CT	GA	TT	AA
28	244	F	164	57	21.19	18	6.27	GA	GT	CT	GA	TT	AA
29	245	M	173	52	17.37	19	7.81	GA	TT	CT	GA	CT	AA
30	246	F	166	64	23.23	18	7.937	GA	GG	CT	GA	CT	AA
31	247	F	154	50	21.08	19	8.108	AA	TT	CT	GA	CT	GA
32	249	F	157	55	22.31	18	9.02	GG	GG	CT	GA	CC	AA
33	250	F	155	54	22.48	19	7.61	GA	GT	TT	AA	TT	AA
34	251	F	168	60	21.26	20	6.384	AA	TT	CT	GA	CT	AA
35	252	F	164	56	20.82	19	8.542	GG	GG	CT	GA	TT	AA
36	254	F	164	56	20.82	19	8.58	AA	GT	CC	GG	CT	GG
37	256	F	170	58	20.07	18	8.812	GA	GT	CT	AA	TT	AA
38	257	F	156	52	21.37	19	9.933	GA	GT	CT	GG	TT	AA
39	259	M	165	49	18.00	18	8.21	GA	GT	CT	GA	TT	AA
40	260	M	170	47	16.26	18	8.21	GA	GT	CT	GA	TT	AA
41	261	F	168	54	19.13	19	8.734	GA	GT	TT	AA	TT	AA
42	262	F	161	54	20.83	19	7.579	GA	GT	TT	AA	CT	AA
43	265	F	159	54	21.36	20	8.95	AA	TT	CT	GA	TT	GA
44	266	F	156	45	18.49	18	7.1	AA	GT	CT	GA	CT	GA
45	267	F	157	44	17.85	19	6.77	GA	GT	CT	GA	CT	GA
46	268	F	155	48	19.98	19	6.584	AA	TT	TT	AA	TT	AA

47	269	F	151	37	16.23	19	7.27	AA	TT	CT	GA	CT	GA
48	270	F	151	48	21.05	19	6.169	GA	GT	CT	GG	TT	AA
49	272	F	167	57	20.44	20	13.1	GA	GT	TT	AA	TT	AA
50	274	F	150	50	22.22	19	7.674	AA	GT	TT	AA	CT	GA
51	276	F	160	59	23.05	19	8.63	GA	GT	CC	GG	CT	GA
52	277	F	170	62	21.45	17	6.36	GA	TT	CT	GG	CC	GA
53	279	F	170	68	23.53	17	8.55	GA	GT	CT	GA	CT	GA
54	281	F	157	60	24.34	18	9.53	AA	TT	CT	GA	CT	GA
55	284	F	158	55	22.03	18	11.1	AA	TT	CT	GA	CC	GG
56	285	F	157	67	27.18	20	6.77	GG	GT	TT	AA	TT	AA
57	286	M	172	84	28.39	24	6.03	AA	GT	TT	AA	CT	GA
58	287	F	173	74	24.73	17	9.68	GG	GT	CC	GG	CT	GA
59	290	F	164	80	29.74	20	8.21	AA	GT	CC	GG	CC	GG
60	292	F	157	47	19.07	19	8.285	GA	GT	CC	GG	CC	GG
61	293	M	175	90	29.39	19	8.21	AA	TT	CT	GA	CC	GA
62	294	F	165	52	19.10	24	5.522	AA	TT	TT	AA	CT	AA
63	295	F	160	64	25.00	23	9.08	GA	TT	CC	GG	CT	GA
64	296	F	161	60	23.15	18	8.58	GA	GT	CC	GG	CT	GA
65	297	F	160	56	21.88	18	8.394	AA	TT	TT	AA	TT	AA
66	298	M	166	70	25.40	20	7.81	GA	TT	TT	AA	TT	AA
67	299	F	168	55	19.49	19	6.714	GA	GG	TT	AA	CT	AA
68	300	F	164	60	22.31	21	7.156	GA	GG	CC	GG	CC	AA
69	302	F	164	52	19.33	20	9.347	GA	GG	CC	GG	CT	GA
70	303	F	170	60	20.76	19	9.053	GA	GT	TT	AA	CT	AA
71	306	F	168	53	18.78	23	9.09	GA	GT	TT	AA	CT	GA

72	307	F	160	59	23.05	19	6.508	GA	GT	CT	GG	CT	GA
73	308	F	171	57	19.49	19	7.516	GG	GT	CT	GG	CC	GG
74	310	F	160	47	18.36	18	5.52	GA	GT	CC	GG	CC	GG
75	312	F	155	55	22.89	19	6.287	GG	GG	CC	GG	CT	GA
76	313	F	165	48	17.63	19	6.61	GA	GT	TT	GA	TT	AA
77	315	F	167	58	20.80	20	6.903	GA	GT	CT	GA	CT	GA
78	318	F	152	55	23.81	23	6.821	GA	GT	CT	GA	CT	GA
79	319	M	173	63	21.05	25	9.085	GG	GG	CT	GA	CT	GA
80	321	F	156	41	16.85	23	8.42	AA	TT	CT	GA	CT	AA
81	323	M	170	70	24.22	19	7.81	GG	GG	CC	AA	CT	GA
82	327	M	178	63	19.88	19	6.77	AA	TT	CT	GA	CT	GA
83	329	M	173	67	22.39	19	7.27	AA	GT	TT	AA	TT	GA
84	330	M	179	72	22.47	19	6.77	GA	GT	CC	GG	CC	GA
85	331	M	165	69	25.34	21	7.81	GG	GG	CC	GG	CT	GA
86	333	M	172	67	22.65	20	7.45	AA	TT	CT	GA	CC	GG
87	334	M	173	80	26.73	23	8.42	AA	TT	CT	GA	CC	GA
88	335	M	167	62	22.23	22	7.1	GG	GG	CT	AA	TT	AA
89	336	F	164	54	20.08	19	6.714	GA	GT	CT	GA	CC	GA
90	337	M	173	73	24.39	22	8.42	AA	TT	CT	GA	CT	GA
91	338	M	176	60	19.37	21	8.01	AA	GT	CC	GG	CT	GA
92	339	F	167	49	17.57	18	8.42	GA	GT	CT	GA	CC	GG
93	340	F	155	50	20.81	21	7.07	GA	GT	CT	GA	CT	GA
94	342	F	157	50	20.28	20	5.522	GA	GT	CC	GG	CT	GA
95	343	F	154	51	21.50	20	19.86	GA	GT	TT	AA	TT	AA
96	344	M	180	65	20.06	22	8.21	GA	TT	CT	GA	CT	AA

97	346	M	182	66	19.93	18	7.27	GA	GT	CT	GA	CT	AA
98	347	F	161	70	27.01	19	7.45	GA	GT	CT	GA	CC	GG
99	348	M	171	68	23.26	23	7.45	GA	GG	CC	GG	TT	AA
100	349	M	171	79	27.02	19	7.63	GA	GG	CT	GA	CC	GA
101	350	M	176	60	19.37	18	6.03	AA	TT	CC	GG	CT	GA
102	351	F	171	54	18.47	18	6.93	AA	TT	TT	AA	TT	AA
103	352	F	158	57	22.83	19	6.584	AA	GT	CC	GG	CT	GA
104	353	F	160	48	18.75	19	5.366	AA	TT	CT	GA	CT	AA
105	354	M	166	54	19.60	19	6.17	GA	TT	CT	GA	CT	GA
106	357	M	171	65	22.23	20	7.63	AA	TT	CT	GG	CC	GG
107	359	M	175	75	24.49	18	6.93	AA	TT	TT	AA	TT	AA
108	361	F	160	58	22.66	18	7.579	AA	GT	TT	AA	TT	AA
109	362	M	175	80	26.12	21	5.9	GA	GT	CC	GG	TT	AA
110	363	M	187	89	25.45	21	5.52	GA	GT	TT	AA	CC	GA
111	364	M	174	55	18.17	18	5.9	AA	TT	TT	AA	CT	AA
112	365	M	171	55	18.81	19	7.45	AA	TT	CT	GA	CT	GA
113	367	F	150	47	20.89	19	6.958	GA	TT	CT	GA	TT	AA
114	368	M	175	69	22.53	20	8.42	GG	GG	CT	GA	CT	GA
115	369	F	160	53	20.70	19	9.468	GA	GT	CC	GG	CC	GA
116	370	M	174	58	19.16	22	6.77	GA	GT	CT	GA	CC	GG
117	374	M	180	70	21.60	19	7.1	GG	GG	CC	GG	CT	GA
118	375	F	165	70	25.71	17	4.18	AA	TT	TT	AA	CT	AA
119	376	F	160	55	21.48	19	12.03	AA	TT	CT	GA	TT	AA
120	378	F	155	50	20.81	17	14.5	GG	GG	CC	GG	CC	GG
121	379	F	162	47	17.91	18	7.63	GA	TT	CT	GA	TT	AA

122	380	M	175	72	23.51	18	7.27	GA	GG	CT	GA	CT	GA
123	381	M	175	60	19.59	20	7.27	GA	GT	CC	GG	CT	GA
124	382	M	170	52	17.99	18	5.64	GA	TT	CT	GA	TT	AA
125	383	M	170	68	23.53	23	6.46	AA	TT	TT	AA	TT	AA
126	384	M	165	65	23.88	22	7.1	AA	TT	CC	GG	TT	AA
127	386	M	163	61	22.96	21	6.77	GA	GT	CT	GA	TT	AA
128	387	F	169	56	19.61	18	15.14	GA	GT	CT	GA	TT	AA
129	389	M	170	68	23.53	21	7.45	AA	TT	TT	AA	CC	GA
130	390	M	182	81	24.45	22	7.27	AA	TT	TT	AA	TT	AA
131	391	M	184	84	24.81	21	7.81	GG	GT	CT	GA	CT	GA
132	392	F	170	75	25.95	18	8.36	AA	TT	TT	AA	TT	AA
133	393	M	170	69	23.88	20	7.45	GA	GT	CC	GG	CC	GA
134	394	M	171	62	21.20	27	5.9	GA	GT	CC	GG	CT	GA
135	395	F	163	48	18.07	18	6.4	AA	GT	TT	AA	TT	AA
136	396	M	175	68	22.20	19	5.39	AA	TT	CC	GG	CT	GG
137	397	M	167	58	20.80	19	7.27	GA	GT	CC	GG	CT	GA
138	398	F	164	68	25.28	21	8.88	GA	GT	CT	GA	CT	GA
139	399	M	169	70	24.51	19	7.1	GA	GT	CT	GA	CT	GA
140	400	M	170	55	19.03	24	6.93	GA	GT	TT	AA	CC	GA
141	402	M	180	68	20.99	22	6.31	GA	GT	CT	GA	CT	AA
142	403	M	178	72	22.72	22	4.74	AA	TT	TT	AA	CC	AA
143	404	M	180	75	23.15	19	5.16	GA	GT	TT	AA	CT	AA
144	406	M	168	58	20.55	19	6.46	AA	TT	CC	GG	CC	GG
145	407	M	172	72	24.34	18	6.03	GA	GG	CT	GA	CC	GG
146	408	M	161	55	21.22	18	5.52	AA	TT	TT	AA	CT	GA

147	410	F	157	42	17.04	19	1.1	AA	GT	CT	GA	TT	AA
148	412	M	175	67	21.88	18	7.63	AA	TT	CT	GA	CC	GA
149	413	F	158	45	18.03	18	9.84	AA	TT	CT	GG	TT	AA
150	414	M	175	63	20.57	17	5.28	AA	TT	CT	GA	TT	AA
151	416	M	169	54	18.91	19	5.39	AA	TT	CC	GA	CT	GA
152	417	F	155	42	17.48	18	8.61	GA	TT	CT	GG	CT	GA
153	418	M	172	58	19.61	18	9.76	AA	GT	CC	GG	CT	GA
154	419	M	173	80	26.73	19	12.1	GA	GT	CC	GG	CT	GA
155	603	F	162	53	20.20	19	12.03	GG	GT	CC	GG	CT	AA
156	604	M	160	44	17.19	19	10.2	AA	TT	CT	GA	CT	AA
157	605	F	153	50	21.36	19	10.74	GG	GG	CT	GA	TT	GA
158	606	M	170	71	24.57	20	8.88	GG	TT	TT	AA	TT	AA
159	607	F	160	61	23.83	19	8.616	AA	TT	CT	GA	CT	GA
160	608	M	186	65	18.79	18	8.95	GG	GT	TT	AA	CT	GA
161	609	M	175	75	24.49	19	8.68	AA	GT	TT	AA	TT	AA
162	611	F	160	59	23.05	19	13.49	AA	TT	CT	GA	CT	GA
163	612	F	164	52	19.33	19	9.836	GA	GG	TT	AA	CT	GA
164	613	F	161	63	24.30	19	8.738	GA	TT	CC	GG	CT	AA
165	614	F	151	56	24.56	19	5.365	AA	TT	CT	GA	TT	AA
166	615	M	172	70	23.66	19	8.05	GG	GT	CT	GA	CT	GA
167	617	F	160	56	21.88	18	5.365	GG	GT	CT	GA	CC	GG
168	618	M	178	75	23.67	22	8.48	GG	TT	CT	GA	CT	GA
169	619	F	165	56	20.57	19	11.02	AA	GT	CC	GG	CC	GA
170	620	F	156	45	18.49	18	10	GG	GT	CC	GG	CC	GG
171	621	M	177	49	15.64	20	15.4	GA	GT	CT	GA	TT	AA

172	622	F	157	65	26.37	19	13.2	GG	GT	TT	AA	CT	GA
173	623	F	155	52	21.64	21	9.19	GA	GT	CC	GG	CT	GA
174	624	F	160	60	23.44	18	10.74	GA	TT	CC	GG	CC	GA
175	625	F	160	54	21.09	19	11.81	AA	TT	CC	GG	CC	GG
176	627	M	185	100	29.22	19	10.8	GA	TT	CT	GA	CT	AA
177	629	M	162	48	18.29	20	8.17	AA	TT	CC	GG	CT	GA
178	630	M	176	51	16.46	19	9.31	GA	GT	CT	GA	TT	AA
179	631	M	174	68	22.46	25	7.49	AA	GT	CC	GG	CC	GG
180	632	F	155	53	22.06	29	9.539	AA	GT	CT	GA	TT	AA
181	633	F	160	67	26.17	19	7.99	GG	GT	TT	GA	TT	AA
182	635	F	160	55	21.48	19	11.5	GA	GT	CC	GG	CC	GG
183	636	M	188	80	22.63	19	6.57	GA	GT	CT	GA	TT	AA
184	637	M	175	63	20.57	19	8.61	GA	GT	TT	AA	TT	AA
185	638	F	172	60	20.28	19	12.37	AA	TT	CT	GA	CC	GA
186	639	F	158	49	19.63	19	15.14	AA	TT	CT	GA	TT	AA
187	640	F	150	56	24.89	19	21.33	AA	TT	TT	AA	TT	AA
188	641	M	183	84	25.08	19	12.2	GG	GG	TT	AA	CC	AA
189	642	M	179	65	20.29	18	9.68	AA	TT	TT	AA	TT	AA
190	644	M	170	53	18.34	19	6.98	GG	GG	CT	GA	CC	GA
191	646	F	163	60	22.58	19	15.31	GG	GG	CT	GA	TT	AA
192	647	M	182	77	23.25	19	5.84	AA	GT	TT	AA	CT	AA
193	648	F	155	38	15.82	19	8.23	GA	GT	TT	AA	TT	AA
194	649	M	173	78	26.06	20	9.16	GA	GT	TT	AA	TT	AA
195	650	F	150	45	20.00	21	16.39	GG	GG	TT	AA	TT	AA
196	651	M	171	60	20.52	18	10.4	GA	GT	CT	GA	CC	GG

197	652	F	165	58	21.30	22	3.401	AA	TT	CC	GG	CT	GA
198	653	F	160	47	18.36	19	10.5	GA	GT	TT	AA	TT	AA
199	655	F	155	50	20.81	19	10.65	GA	TT	CC	GG	CT	GA
200	656	F	167	58	20.80	19	8.863	AA	GT	CC	GG	CC	GG
201	657	F	164	63	23.42	19	5.792	AA	GT	TT	AA	CT	AA
202	658	F	163	63	23.71	19	10.07	GA	GT	TT	AA	CT	AA
203	659	F	161	60	23.15	19	16.59	AA	TT	CT	GA	CT	GA
204	660	F	168	69	24.45	19	7.006	GG	TT	TT	AA	CC	GG
205	661	F	162	44	16.77	19	9.84	AA	TT	CT	GA	TT	AA
206	662	M	176	75	24.21	18	10.6	AA	TT	CT	GA	CT	AA
207	663	M	164	50	18.59	22	6.48	AA	GG	TT	AA	TT	AA
208	664	F	161	59	22.76	19	12.72	AA	TT	CC	GG	CT	GA
209	675	F	165	60	22.04	19	8.497	AA	TT	CT	GA	CT	AA
210	678	F	162	52	19.81	18	8.381	GA	GT	CT	GA	CC	GG
211	681	F	160	51	19.92	25	7.048	GA	TT	CT	GA	CT	GA
212	682	F	157	48	19.47	23	7.64	GA	GT	CC	GG	CT	GA
213	683	F	165	54	19.83	30	7.689	GG	GG	TT	AA	TT	AA
214	684	F	165	59	21.67	23	6.35	GA	GT	TT	AA	CT	AA
215	686	F	164	62	23.05	20	7.048	GA	GG	TT	AA	CT	GA
216	687	F	167	58	20.80	19	8.212	GA	TT	TT	AA	TT	AA
217	689	F	158	54	21.63	19	7.449	GA	GT	CT	GA	CT	GA
218	690	F	158	54	21.63	19	11.3	AA	GT	CT	GA	TT	AA
219	691	M	180	66	20.37	18	10.2	GA	GT	CT	GA	CC	GA
220	692	F	164	53	19.71	20	7.222	AA	TT	TT	AA	TT	AA
221	693	M	173	76	25.39	20	8.61	AA	TT	CC	GG	CT	GA

222	694	M	165	65	23.88	18	7.49	AA	TT	TT	AA	CC	GA
223	695	F	168	59	20.90	19	7.091	GA	GG	TT	AA	CT	GA
224	696	F	167	65	23.31	20	9.123	GA	GT	CC	GG	CC	GG
225	712	M	173	74	24.73	18	7.94	GG	GG	TT	AA	TT	AA
226	713	F	154	57	24.03	22	10.74	AA	TT	CT	GA	CT	GA
227	715	F	167	55	19.72	19	7.449	AA	GT	CT	GA	TT	AA
228	716	F	160	60	23.44	19	6.245	GA	GT	CT	GA	CC	GA
229	717	M	156	50	20.55	20	9.16	GA	TT	CC	GG	CT	GA
230	719	F	153	46	19.65	21	7.178	GG	GG	CT	GA	CT	GA
231	720	F	162	52	19.81	20	9.258	AA	TT	CT	GA	CC	GG
232	721	M	160	50	19.53	20	9.61	GG	GG	CT	GA	TT	AA
233	722	F	160	66	25.78	23	10.4	AA	TT	CC	GG	CT	AA
234	724	F	165	66	24.24	23	7.739	GG	GG	CC	GG	TT	AA
235	725	F	163	50	18.82	24	8.8	GA	GG	CT	GA	CC	GG
236	726	F	163	60	22.58	22	5.615	GA	GT	CT	GA	CC	GA
237	727	F	173	65	21.72	24	7.891	GA	GG	CT	GA	CT	GA
238	728	F	156	52	21.37	28	7.222	AA	GT	CT	GA	TT	AA
239	729	F	160	43	16.80	18	9.61	GA	GT	CT	GA	CC	GG
240	730	F	171	66	22.57	19	4.674	AA	GT	CT	GA	CC	GA
241	732	F	168	60	21.26	19	6.458	AA	TT	CC	GG	CC	AA
242	733	F	157	60	24.34	23	6.077	AA	GT	CC	GG	CT	GA
243	735	F	163	45	16.94	19	7.76	GG	GG	CT	GA	CT	AA
244	736	F	158	55	22.03	19	5.084	GA	GT	CC	GG	CC	GG
245	737	M	172	62	20.96	17	8.75	GG	GT	CT	GA	CT	GA
246	738	F	150	42	18.67	24	6.458	AA	GT	CC	GG	CT	AA

247	739	F	160	75	29.30	24	8.42	AA	TT	CC	GG	CT	GA
248	740	F	161	50	19.29	23	8.556	GA	GG	CT	GA	CT	GA
249	741	F	163	67	25.22	23	8.68	GA	GT	TT	AA	TT	AA
250	742	F	164	47	17.47	22	9.53	GA	GT	CT	GA	TT	AA
251	743	F	173	63	21.05	23	11.4	GA	GT	CC	GG	CC	GG
252	745	F	158	50	20.03	21	6.044	GG	GT	TT	AA	CT	AA
253	746	F	166	60	21.77	21	5.209	AA	GT	CC	GG	CT	GA
254	747	F	163	60	22.58	21	7.178	AA	GT	CT	GA	CT	GA
255	749	F	150	48	21.33	21	7.134	GA	GT	CT	GA	TT	AA
256	751	F	152	40	17.31	21	9.02	GA	GT	CT	GA	CT	GA
257	752	M	177	55	17.56	20	7.94	AA	TT	CT	GG	CT	GA
258	753	F	159	48	18.99	21	4.695	GA	GT	CC	GG	CT	GA
259	754	F	160	75	29.30	20	9.61	AA	TT	CT	GA	CT	AA
260	755	M	172	67	22.65	22	7.44	GA	TT	CT	GA	TT	AA
261	756	F	166	48	17.42	22	9.84	AA	TT	CT	GA	CT	AA
262	758	F	152	43	18.61	20	7.357	GG	GG	CT	GA	CT	AA
263	759	F	148	45	20.54	19	5.823	GA	TT	TT	AA	CT	AA
264	760	M	175	75	24.49	24	5.19	GA	GT	CT	GA	TT	AA
265	761	F	166	55	19.96	21	12.97	GG	GG	CC	GG	CC	GG
266	762	M	171	71	24.28	20	7.33	AA	TT	TT	AA	CT	AA
267	763	F	155	54	22.48	22	5.183	GA	GG	CT	GA	CT	GA
268	764	F	160	66	25.78	20	8.05	AA	TT	CC	GG	CT	GA
269	765	F	158	48	19.23	20	7.091	GA	GT	CT	GG	CC	GA
270	766	F	158	79	31.65	25	6.31	GA	GT	CT	GA	CC	GA
271	767	F	160	51	19.92	20	7.266	GG	GT	TT	AA	CT	AA

272	768	F	157	48	19.47	20	4.509	GA	GT	CC	GG	CT	GA
273	769	F	150	48	21.33	20	5.338	AA	TT	CT	GA	CC	GA
274	771	M	174	62	20.48	23	14.8	AA	TT	CC	GG	CT	GA
275	772	M	172	67	22.65	21	9.23	AA	TT	CC	GG	CC	GG
276	773	F	162	51	19.43	21	5.947	GA	GT	CT	GA	CT	GA
277	774	F	156	60	24.65	22	4.549	GA	GG	CC	GG	CC	GG
278	775	M	182	75	22.64	21	7.23	GG	GG	CT	GA	CC	GA
279	776	M	173	70	23.39	19	4.75	GA	TT	TT	AA	TT	AA

Note. F – females; M – males.

Table S3. Allele frequencies of studied SNP-markers of *UCP1, UCP2* and *UCP3* genes in the Yakut population

No	SNP	Gene	Genotypes (n)			Allele frequency		HWE[1]
1	rs1800592	*UCP1*	AA (109)	AG (126)	GG (44)	A 0.616	G 0.384	0.468
2	rs3811787		TT (101)	TG (132)	GG (46)	T 0.599	G 0.401	0.356
3	rs659366	*UCP2*	TT (74)	TC (134)	CC (71)	T 0.505	C 0.495	0.593
4	rs660339		AA (75)	AG (123)	GG (81)	A 0.489	G 0.511	0.05
5	rs1800849	*UCP3*	TT (85)	CT (127)	CC (67)	T 0.532	C 0.468	0.155
6	rs2075577		AA (124)	AG (120)	GG (35)	A 0.659	G 0.341	0.499

Note. [1] – p-values of deviation from Hardy–Weinberg Equilibrium (HWE).

Table S4. ANOVA analysis of plasma irisin levels depending on genotypes in the Yakut's with normal weight (n=214)

Gene, SNP	Genotypes<br>Mean ± std.dev			F; p
UCP1, rs1800592	GG	AG	AA	
F	9.15±3.25	8.13±2.45	8.5±3.35	F=1.011; *p*=0.366
M	8.23±0.78	7.14±1.39	7.48±1.94	F=2.154; *p*=0.124
UCP1, rs3811787	TT	GT	GG	
F	8.95±3.48	7.98±2.52	8.73±2.91	F=1.726; *p*=0.182
M	7.47±1.98	7.5±1.37	7.63±1.02	F=0.04; *p*=0.96
UCP2, rs659366	CC	CT	TT	
F	8.38±2.66	8.27±2.66	8.76±3.67	F=0.331; *p*=0.719
M	7.68±2.1	7.66±1.36	7.12±1.43	F=0.828; *p*=0.441
UCP2, rs660339	AA	AG	GG	
F	8.76±3.62	8.34±2.8	8.25±2.5	F=0.343; *p*=0.71
M	7.15±1.37	7.6±1.44	7.8±2.09	F=0.884; *p*=0.418
UCP3, rs1800849	CC	CT	TT	
**F**	**8.47±2.6**	**7.73±2.24**	**9.47±3.77**	**F=4.8; *p*=0.01**
M	7.49±1.39	7.66±1.85	7.27±1.36	F=0.36; *p*=0.699
UCP3, rs2075577	GG	GA	AA	
F	9.22±2.6	7.86±2.47	8.71±3.34	F=2.178; *p*=0.117
M	7.43±1.57	7.8±1.67	7.12±1.53	F=1.165; *p*=0.318

Note. F – females; M – males.

Table S5. Analysis of irisin levels, weight, height a BMI depending on genotypes in the women's SNP rs1800849 gene *UCP3*

Parameters, genotypes	Mean ± std.dev	*p*
Irisin (n=144)		
TT vs CC	9.47 ± 3.77 vs 8.47 ± 2.6	0.324
**TT vs CT**	**9.47 ± 3.77 vs 7.73 ± 2.24**	**0.01**
CC vs CT	8.47 ± 2.6 vs 7.73 ± 2.24	0.195
Weight (n=185)		
**TT vs CC**	**53.61 ± 6.97 vs 58.27 ± 11.56**	**0.03**
TT vs CT	53.61 ± 6.97 vs 55.51 ± 8.19	0.11
CC vs CT	58.27 ± 11.56 vs 55.51 ± 8.19	0.415
Height (n=185)		
**TT vs CC**	**160.04 ± 5.85 vs 163.18 ± 5.9**	**0.03**
TT vs CT	160.04 ± 5.85 vs 160.37 ± 5.51	0.8
**CC vs CT**	**163.18 ± 5.9 vs 160.37 ± 5.51**	**0.02**
BMI (n=185)		
TT vs CC	20.94 ± 2.45 vs 21.82 ± 3.6	0.443
TT vs CT	20.94 ± 2.45 vs 21.53 ± 2.65	0.229
CC vs CT	21.82 ± 3.6 vs 21.53 ± 2.65	0.773

Table S6. Frequencies of the T allele of the rs1800849 polymorphism (*UCP3*) in twelve Asian populations living in different climatic zones

Populations	n	Climatic zones	Frequency of the T allele (CI)	Total frequency of the T allele (CI)	*p*
"North Asia"					0.01
Chukchi (CHU) [33]	95	subarctic	0.33 (0.25-0.424)	0.45 (0.42-0.484)	
Yakuts (YAK) [this study]	279	temperate	0.53 (0.479-0.587)		
Koryaks (KOR) [33]	89	temperate	0.53 (0.432-0.622)		
Kets (KET) [33]	48	temperate	0.35 (0.24-0.485)		
Khanty (KHA) [33]	95	temperate	0.52 (0.428-0.613)		
Buryats (BUR) [33]	95	temperate	0.48 (0.387-0.613)		
Nivkhs (NIV) [33]	95	temperate	0.22 (0.152-0.306)		
"South Asia"					
Chinese (CHB) [32]	103	temperate	0.32 (0.242-0.408)	0.28 (0.244-0.316)	
Japanese (JPT) [32]	104	subtropical	0.30 (0.227-0.39)		
Chinese (CHS) [32]	105	subtropical	0.26 (0.191-0.346)		
Chinese (CDX) [32]	93	subequatorial	0.23 (0.16-0.318)		
Vietnamese (KHV) [32]	99	subequatorial	0.27 (0.198-0.36)		
Total	N=1300				

Note. n – number of individuals; N – total number of individuals; CI – confidence interval; YAK –Yakuts; the data is taken from Stepanov et al., [33]: CHU – Chukchi, KOR – Koryaks, KET – Kets, KHA – Khantys, BUR – Buryats, NIV – Nivkhs. The data is taken from the open database "1000 Genomes Project" [32]: CHB – Han Chinese, Benjing, China; JPT – Japanese in Tokyo, Japan; CHS – Southern Han Chinese, China; CDX – Chinese Dai in Xishuangbanna, China; KHV – Kinh Ho Chi Minh City, Vietnam.
